# Top-Down Modulations from Dorsal Stream in Lexical Recognition: An Effective Connectivity fMRI Study

**DOI:** 10.1371/journal.pone.0033337

**Published:** 2012-03-13

**Authors:** Yuan Deng, Ruifang Guo, Guosheng Ding, Danling Peng

**Affiliations:** 1 Key Laboratory of Behavioral Science, Institute of Psychology, Chinese Academy of Science, Beijing, China; 2 State Key Lab of Cognitive Neuroscience and Learning, Beijing Normal University, Beijing, China; 3 Changzhi Medical College, Changzhi, Shanxi, China; University of Maryland, College Park, United States of America

## Abstract

Both the ventral and dorsal visual streams in the human brain are known to be involved in reading. However, the interaction of these two pathways and their responses to different cognitive demands remains unclear. In this study, activation of neural pathways during Chinese character reading was acquired by using a functional magnetic resonance imaging (fMRI) technique. Visual-spatial analysis (mediated by the dorsal pathway) was disassociated from lexical recognition (mediated by the ventral pathway) via a spatial-based lexical decision task and effective connectivity analysis. Connectivity results revealed that, during spatial processing, the left superior parietal lobule (SPL) positively modulated the left fusiform gyrus (FG), while during lexical processing, the left SPL received positive modulatory input from the left inferior frontal gyrus (IFG) and sent negative modulatory output to the left FG. These findings suggest that the dorsal stream is highly involved in lexical recognition and acts as a top-down modulator for lexical processing.

## Introduction

According to the well-known visual domain hypothesis [1], when humans process visual stimuli, visual information first arrives at the occipital lobe of the brain, and is then processed via the dorsal and ventral visual pathways. In the ventral pathway, visual information is projected to the temporal lobe for the identification of visual features (“what”). In the dorsal pathway, visual information is projected to the parietal lobe for the spatial/motion analysis (“where”) [2].

Consistent with the what/where visual pathways, the ventral and dorsal visual streams also play important roles during reading. According to a modern vision of the cortical networks for reading [3], the ventral pathway (e.g., occipito-temporal region) is responsible for identifying the word form while the dorsal pathway (e.g., posterior parietal region) plays a role in top-down attention and serial reading. This notion is supported by converging evidence from brain imaging studies. For example, in ventral circuits, the left fusiform gyrus [FG, Broadmans area(BA) 37/19)] performs pattern-based visual analysis specific to a word or word-like stimulus [4]–[9]. In dorsal circuits, evidence from both normal and abnormal reading suggests that the posterior parietal region is involved in word and non-word reading [10], as well as letter position encoding [11]–[13]. Impaired function in this region may lead to dyslexia [14]–[17].

Despite the vast amount of knowledge about the ventral and dorsal streams, little is known about how these two pathways interact, especially in word reading. Evidence from electrophysiological studies in monkeys suggests that the dorsal stream interacts with the frontal region of the brain and modulates the ventral stream to assist in visual object recognition [18]–[20]. Similar evidence has also been found in humans [21]–[24], emphasizing the importance of top-down feedback in object recognition. In terms of lexical processing, some researchers suggest that the regions in the dorsal pathway may be involved in sequentially allocating attention along the letters [14]–[15], [25] and providing top-down feedback to the ventral pathway for letter identification [26]. However, others have found that ventral regions connect to the dorsal area of the brain in a feed-forward way for lexical recognition [10], [27]–[28]. Further investigation is necessary to clarify this inconsistency.

In the current study, we examined interactions between the dorsal and ventral pathways in lexical recognition and explored how these interactions respond to different cognitive demands. To accomplish this, we adopted a spatial-based lexical decision task and took advantage of the uniqueness of Chinese characters. Generally, Chinese characters consist of one or more radicals (components). The radicals have specific positions in a given character (i.e., left, right, top or bottom of the character) [29]–[30]. In rare cases, changing the position of radicals of one character (e.g., 呆, /dai1/, meaning “stupid”) creates another meaningful characters (e.g., 杏, /xing4/, meaning “almond”). However, for most Chinese characters, changing the position of the radicals will generate a pseudo-character.

In this study, participants were presented three kinds of stimuli (conditions): 1) true characters (TC); 2) pseudo-characters whose radicals could be rearranged to create a true character (radical-rearranged true characters, RTC); 3) pseudo-characters whose radicals could not be rearranged in any way to create a true character (radical-rearranged pseudo-characters, RPC). The task was to judge whether the radicals of the stimulus could form a true character, regardless of whether this required rearrangement. Because conditions 1 and 2 could belong to the mental lexicon of true characters, while condition 3 could not. So, processing of lexical, which is believed to be mediated by the ventral pathway, was revealed by comparing conditions 1 and 2 to condition 3. On the other hand, conditions 2 and 3 require mental rearrangement of the radicals' positions, while condition 1 does not. Thus, spatial processing, which is believed to be mediated by the dorsal pathway, was revealed by comparing conditions 2 and 3 to condition 1.

Neural activations during the character decision task were acquired by using functional magnetic resonance imaging (fMRI). Effective connectivity analysis was employed, which could reveal intrinsic connections between regions despite different task requirements and modulation effects of external factors [31], [32]. This method allowed us to examine the connections between the dorsal and ventral pathways and to disassociate the modulatory effects of different cognitive processes (lexicality versus spatiality) on these connections.

To our knowledge, this is the first report of such an approach to explore the functions and interactions of dorsal and ventral pathways. If the dorsal stream is involved in top-down modulation, we would predict the existence of an intrinsic connection from the dorsal pathway to the ventral pathway and a direct impact of the ventral stream in lexical recognition. We would also predict that connection patterns between the two streams would be modulated by the different cognitive demands.

## Methods

### Participants

Participants included 12 adults (5 males and 7 females, aged 18 to 26 years,) who were undergraduate or graduate students at Beijing Normal University. All participants were right-handed and had normal hearing and normal or corrected-to-normal vision. None of the participants had any form or history of reading/learning difficulties. Informed written consent was obtained from the participants before the experiment. This study was approved by the Institutional Review Board of the State Key Laboratory of Cognitive Neuroscience and Learning at Beijing Normal University.

### Materials

**Figure 1:**
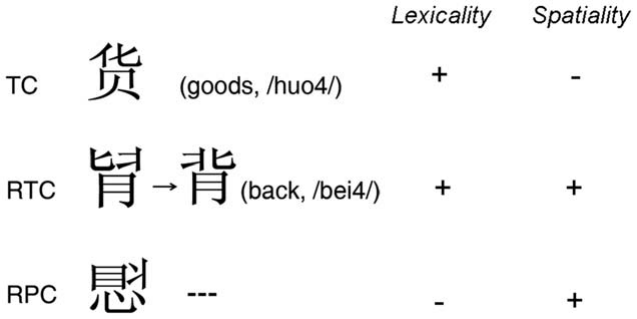
Examples of stimuli. TC, true character; RTC, radical-rearranged true characters; RPC, radical-rearranged pseudo-characters. For RTC, the second character is the true character, created by reversing the upper two radicals. English meaning and Chinese pronunciation (pinyin) for true characters are displayed in parenthesis. “+” and “−” indicate higher or lower involvements of factors.

Three types of stimuli (conditions) were presented, as shown in Figure 1: 1) TC, 2) RTC, and 3) RPC. All stimuli consisted of three radicals. RTC stimuli were created by re-arranging the position of any two radicals in a true character. For example, by switching the position of two upper radicals, the RTC stimulus can be transformed into a true character (背, meaning “back”). For the RPC stimuli, no combinations of the three radicals could create a true character.

The task involved judging whether the radicals of each stimulus could form a true character, regardless of whether they would need to be arranged. Radicals of TC and RTC stimuli can compose a true character (lexical effect) while radicals of RPC cannot. So, responses to conditions 1 and 2 should be “Yes” while response to condition 3 should be “No”. On the other hand, radicals of RTC and RPC stimuli require greater spatial manipulation (spatial effect). Both lexicality and spatiality effects are illustrated in Figure 1, in which “+” and “−” indicate high and low involvement of factors in each of the three conditions.

A total of 135 stimuli were used, including 45 stimuli for each condition. TC and RTC had similar word frequencies (*p* = 0.945), and the visual complexity of characters, indicated by the number of strokes, was balanced across the three types of stimuli (*p* = 0.448).

### fMRI procedure

During fMRI scanning, an event-related design was adopted, and all trials were presented in a pseudorandom sequence. For each trial, a fixation point was presented for 500 milliseconds, followed by a visual stimulus for 1500 milliseconds. The interstimuli interval (ISI) ranged from 4 to 8 seconds. Participants were asked to determine whether the radicals of each stimulus could compose a true character and to respond using an optical response box. Reaction time was recorded. In addition to the 135 stimuli, 45 null events, in which a blank screen was presented and no response was required, served as a baseline. Three fMRI runs were performed and counterbalanced across participants. The duration of each run was 8 minutes.

### Image Acquisition

All images were acquired using a 1.5 Tesla Siemens Trio scanner. Participants lay in the scanner with their head position secured with a specially designed vacuum pillow, holding an optical response box in their hands. The head coil was positioned over the participants' head. Participants viewed visual stimuli that were projected onto a screen via a mirror attached to the inside of the head coil. For functional imaging , a susceptibility weighted single-shot echo planar imaging (EPI) method was used to measure blood oxygenation level-dependent (BOLD) changes. The following scan parameters were used: TE = 40 msec, flip angle = 90°, matrix size = 64×64, field of view = 23 cm, slice thickness = 6 mm with 1.2 mm interval, number of slices = 24, TR = 2000 msec. In addition, a high resolution, T1 weighted 3D image was acquired [Spoiled Gradient Echo (SPGR), TR = 21 ms, TE = 8 msec, flip angle = 19°, matrix size = 169×256, field of view = 25 cm, slice thickness = 1.7 mm, number of slices = 96].

### fMRI Data Analysis

Data analysis was performed using Statistical Parametric Mapping 2 (SPM2). Functional images were corrected for differences in slice-acquisition time to the middle volume and were realigned to the first volume in the scanning session using affine transformations. No participant had more than 3.0 mm of movement in any plane. Co-registered images were normalized to the MNI average template (12 linear affine parameters for brain size and position, 8 non-linear iterations and 2×2×2 nonlinear basis functions). Statistical analyses were calculated on the smoothed data (6 mm isotropic Gaussian kernel) with a high pass filter (128 seconds cutoff period) in order to remove signal drift, cardiac and respiratory effects, and other low frequency artifacts.

Data from each participant were entered into a general linear model using an event-related analysis procedure. Each individual event was modeled using a canonical hemodynamic response function (HRF). Parameter estimates from contrasts of the canonical HRF in single participant models were entered into random-effects analysis across all participants to determine whether activation during a contrast was significant. First, all conditions were combined and compared to baseline to reveal the overall activation pattern for the cognitive task. Threshold was set at *p*<0.05, FDR-corrected, with a cluster size of 10 or greater. Then, differences between each condition were examined using paired t-tests with a statistical threshold of *p*<0.001, uncorrected, with a cluster size of 10 or greater.

To conduct the connectivity analysis, regions-of-interest (ROIs) were selected as those that showed the highest overlap across the three conditions relative to baseline at group level (*p*<0.05, FDR-corrected) [32]. Three key regions were identified: the left fusiform gyrus (FG, peak: −38, −54, −22), superior parietal lobule (SPL, peak: −24, −66, 56) and the dorsal aspect of inferior frontal gyrus (IFG, peak: −46, 4, 26). The SPM Marsbar toolbox [33] was used to construct ROI regions and to calculate the mean estimate of the percent signal change for each condition. Next, the data for these regions were submitted to a 3 (regions)×3 (conditions) ANOVA analysis.

Finally, the dynamic causal modeling (DCM) tool in SPM2 [31] was used to estimate the effective connectivity between the three ROIs. Based on the group results from random-effect analyses, a DCM model of the three regions was constructed. In addition to these left hemisphere ROIs, several other regions showed overlapping activations across the three conditions, including bilateral middle occipital gyrus, bilateral medial frontal gyrus, bilateral superior frontal gyrus, left precentral gyrus, left postcentral gyrus, left precunes, and right middle frontal gyrus. These regions were not included in the model, as they have not been implicated in previous connectivity analysis of lexical processing [28], [32], [34], and it was therefore impossible to formulate a priori hypotheses of their connectivity.

Regional responses for each subject in each condition were extracted by calculating the principal eigenvariate across all voxels within a 6-mm sphere, centered on the most significantly activated voxel. Subject-specific maxima were defined operationally as the most significantly activated voxels within 10 mm of the group maximum. Data from one subject were excluded because no significant activation cluster was observed within 12 mm from the group-reference voxel in either IFG or SPL. For each DCM model, full and reciprocal connections were specified between FG and SPL and between SPL and IFG. The connection between FG and IFG was not included because previous evidence has demonstrated that FG and IFG are not directly connected during lexical processing, but instead their interactions are mediated via the temporal region [32], [34]. Input conditions were modeled as exerting direct effects on the FG. Two modulation effects were included: 1) lexicality, including TC and RTC conditions, and 2) spatiality, including RTC and RPC conditions. During modeling, three types of parameters were calculated for each subject: 1) intrinsic connections between regions in the absence of modulating experimental effects; 2) both lexical and spatial modulatory effects on intrinsic connections (i.e., the changes in the intrinsic connectivity between regions induced by the experimental design); and 3) input to FG. After modeling, a one-sample t-test was performed for each interregional coupling to determine whether the across-subject mean differed from zero.

## Results

### Behavioral results

The average RTs were: TC, 941±215 msec; RTC, 1412±168 msec; RPC, 1494±253 msec. Error rates were 20%, 41%, and 38%, respectively. One-way ANOVAs reveal significant main effects of stimulus type on RT [F (2,10) = 17.196, *p*<0.005] and error rate [F (2,10) = 29.439, *p*<0.001]. Post-hoc analyses revealed that participants showed significantly faster RT and lower error rate in the TC condition compared to the RTC and RPC conditions (*p's*<0.01), which has similar RTs and error rates (*p's*>0.05). These results suggest that the TC condition was less difficult than the RTC and RPC conditions.

### Imaging results

**Figure 2:**
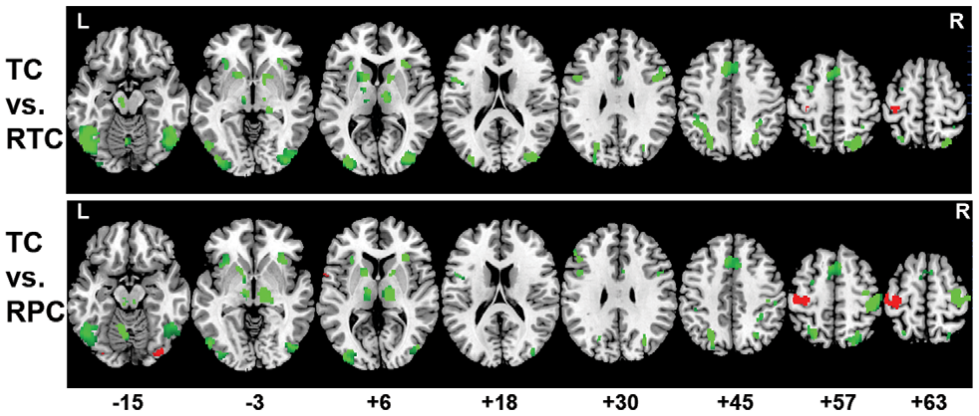
Brain activation maps for differences between conditions. TC, true character; RTC, radical-rearranged true characters; RPC, radical-rearranged pseudo-characters. Green: RTC/RPC greater than TC. Red: TC greater than RTC/RPC.

As shown in Table 1, overall, the cognitive task activated a broad neural network involving many regions related to lexical processing, including the bilateral fusiform gyrus, middle occipital gyrus, inferior/middle/medial frontal gyrus, and left inferior/superior parietal lobule. As shown in Table S1 and Figure 2, compared to TC, both RTC and RPC produced greater activation in the bilateral fusiform gyrus, middle occipital gyrus, inferior/middle frontal gyrus, inferior/superior parietal lobule, thalamus, and putamen. Compared to RTC, TC produced greater activation in the left postcentral gyrus. Unlike RPC, TC produced activations in the left fusiform gyrus and right middle occipital gyrus

**Table 1:** Overlapping activations for all character conditions compared to baseline condition.

Activated regions	BA	Voxels	Z value	x,y,z {mm}
R **culmen**/middle occipital gyrus/fusiform/inferior parietal lobule/superior parietal lobule	18/19/37/7/40	5044	5.7	30 −38 −32
L **culmen**/middle occipital gyrus/fusiform/inferior parietal lobule/superior parietal lobule	18/19/37/7/40	5805	4.71	−38 −56 −32
L **culmen**/L declive/R declive	-	520	4.29	−2 −62 −8
L **putamen**/insula	-	1186	4.27	−20 12 4
R Thalamus	-	505	4.23	10 −16 6
**R superior frontal gyrus**/cingulate gyrus/medial frontal gyrus	8	1597	4	2 16 54
L premotor cortex	6	119	3.99	−26 −6 58
**L insula**/inferior frontal gyrus	9	568	3.86	−40 −4 16
R putamen	-	318	3.78	20 8 8
R inferior frontal gyrus	13	341	3.56	30 24 8
**R middle frontal gyrus**/inferior frontal gyrus	9/45/44	488	3.37	50 12 36
L middle frontal gyrus	8	17	3.34	−54 8 42
L culmen	-	13	3.22	−12 −50 −8
L middle frontal gyrus	46	56	3.22	−50 34 32
R postcentral gyrus	2	82	3.2	44 −24 46
R Uvula	-	19	3.08	8 −76 −44
R middle frontal gyrus	46	114	3.04	42 36 22
L cuneus	17	20	2.92	−14 −80 8
R inferior parietal lobule	40	13	2.91	50 −36 56

L, left hemisphere; R, right hemisphere; BA, Brodmann's areas; Areas in boldface indicate peaks of activation in the clusters. Significance at *p*<0.05, FDR corrected.

**Figure 3:**
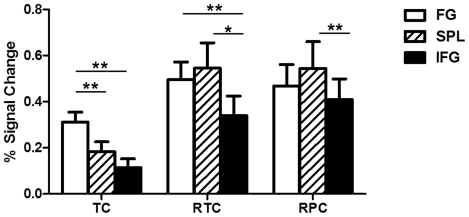
Percentage signal change in ROIs for each condition. TC, true character; RTC, radical-rearranged true characters; RPC, radical-rearranged pseudo-characters. FG, fusiform gyrus; IFG, inferior frontal gyrus; SPL, superior parietal lobule.

Activation intensity for each ROI in each condition are presented in Figure 3. ANOVA revealed significant main effects of condition and region [F_condition_(2,10) = 8.58, *p*<0.001; F_region_(2,10) = 5.64 *p*<0.05], as well as their interaction [F_condition×region_(4,8) = 4.29, *p*<0.05]. Post-hoc analysis revealed higher activation in FG than SPL or IFG in the TC condition, higher activation in FG and SPL than IFG in the RTC condition, and higher activation in SPL than IFG in the RPC condition.

In addition, consistent with the behavioral results, both the RTC and RPC conditions induced greater activation than the TC condition in all ROIs. To evaluate the influence of task difficulty, activation differences between TC and RTC/RPC were correlated with corresponding differences in behavioral performance for each ROI. Correlations were not significant in IFG or FG, but were significant in SPL for the TC versus RTC comparisons [r_(TC vs. RTC)_ = 0.58, *p<.05*]. Therefore, greater activations in SPL may reflect higher spatial processing requirements in the RTC and RPC conditions, but task difficulty may not influence activation of IFG or FG.

**Figure 4:**
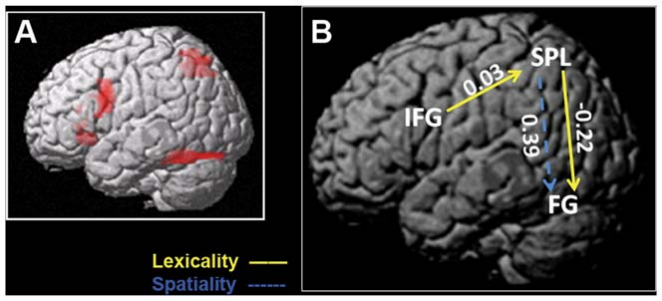
Differential modulatory effects between task demands. A, ROI regions that showed the highest overlap across three conditions comparing to baseline at group level (FDR-corrected, *p*<0.05); B, Yellow solid line arrows indicate influences modulated by lexical judgment, and blue dashed line arrows indicate influences modulated by spatial processing. FG, fusiform gyrus; IFG, inferior frontal gyrus; SPL, superior parietal lobule.

As shown in Figure 4A, although both the ventral and dorsal aspect of inferior frontal gyrus showed significant activation at the group level, activations were consistent across subjects only in the dorsal region, which was the reason for selecting this particular area as the ROI for connectivity analysis. As shown in Table 2 and Figure 4B, the DCM results revealed a significant intrinsic connection from SPL to FG and a tendency for a connection from SPL to IFG. Spatial processing demand significantly positively modulated the connection from SPL to FG, while lexical judgment demand significantly negatively modulated the connection of the SPL to FG and showed a trend to positively modulate in the connection from IFG to SPL. The negative connectivity values for the connection from SPL to FG indicate an inverse modulatory effect (i.e., increases in activity within the SPL reduced activity within the FG and vice versa).

**Table 2:** Intrinsic connections between ROIs and modulatory effects on each connection.

	IFG→SPL	SPL→IFG	SPL→FG	FG→SPL
Intrinsic connection	0.1163	0.0952*	0.6128**	0.0749
*Modulation*				
Lexicality	0.0297*	−0.0345	−0.2225*	0.0254
Spatiality	−0.0043	0.0078	0.3852**	−0.0671

** Significance at *p*<0.0125 (corrected for 4 comparisons);

* Marginally significant tendency at *p*<0.09.

FG, fusiform gyrus; IFG, inferior frontal gyrus; SPL, superior parietal lobule.

## Discussion

These findings demonstrate a role of the left superior parietal lobule in lexical processing, which has important implications for interactions between the dorsal and ventral pathways in reading. Specifically, the left FG showed the greatest activation in lexical recognition (TC condition), while the left SPL and FG showed greater activation with both lexical recognition and spatial processing (RTC and RPC condition). Effective connectivity analysis further demonstrated an intrinsic connection from SPL (dorsal stream) to FG (ventral stream). Finally, spatial processing demand positively modulated the connection from SPL to FG, while lexical processing demand had a negative modulatory effect on this same connection.

A great deal of evidence suggested that regions in the dorsal pathway (e.g., the posterior parietal lobe) are highly involved in lexical recognition. Effective connectivity studies also revealed that dorsal parietal region is a critical part of the pathway for processing both real and pseudo-words in English [10], and dysfunction of the posterior parietal region may partially underlie the reading deficit of dyslexia in English [14], [25]. Neuro-imaging studies in Chinese reading have also suggested that the left superior parietal region plays a critical role in both Chinese character recognition [35]–[37] and learning [38]. The current study directly demonstrated that the dorsal pathway significantly interacts with the ventral pathway and is highly involved in Chinese character recognition.

Previous studies have reported conflicting findings about the specific role of the dorsal pathway and its interaction with the ventral pathway in reading. Some studies suggested that regions in the ventral pathway connect to the regions in the dorsal pathway in a feed-forward way during lexical recognition [10], [27]–[28]. For example, researchers found a significant connection from the left inferior occipital gyrus (LIOG) to the left superior parietal lobule (LSPL) (i.e., “where” pathway) in a chunk decomposition task [28]. In addition, the LIOG→LSPL connection significantly increased when processing real characters and pseudo-characters with tight word form (high perceptual tightness), suggesting involvement of the dorsal parietal region in visual-spatial analysis. However, other researchers suggested that the dorsal pathway plays a role in top-down feedback to the ventral pathway for serial letter-order encoding [12], [14], [25]–[26] and for generation and perception of visual form [39]. Through effective connectivity analyses, our study suggests that there is an intrinsic connection from SPL (dorsal stream) to FG (ventral stream) in the absence of modulating experimental effects, supporting its crucial role in top-down influence to the ventral pathway.

Moreover, the specific function of the connection from SPL (dorsal) to FG (ventral) depends on task demands (i.e., modulatory effects). For spatial-based processing, SPL presented a strong positive top-down modulation of the occipito-temporal region (i.e., an increase in SPL activity leads to an increase in FG activity). When spatial processing was highly involved (RTC and RPC conditions), the left superior parietal region showed greater activation, supporting the dominant role of the dorsal pathway in spatial processing [2].

During lexical recognition, top-down influence exhibited an inhibitory effect (i.e., an increase in SPL activity caused a decrease rate of change in FG activity). Lexical processing also modulated the connection from the dorsal IFG to the SPL. The involvement of the prefrontal region may elucidate the specific role of top-down modulation from SPL to FG. According to previous studies, the dorsal IFG is mainly involved in phonological manipulation [40]–[42] and word retrieval/selection [43]–[45]. Some evidence has also suggested that the fronto-parietal network is responsible for word selection [43], [46]–[49]. In the current study, phonological processing was not likely involved in this task due to the logographic characteristic of Chinese characters and the task requirements (lexical decision), thus the involvement of dorsal IFG is more likely related to controlled semantic retrieval and selection. Word selection likely involves both excitation of intended words and inhibition of unintended words [43]. Therefore, the negative modulatory effect of SPL on FG may be associated with inhibition of the unintended words.

One explanation for the negative modulation effect happening mainly with lexical recognition rather than with spatial processing may have been the task-specific requirements in the current study. Because the character decision task emphasized the lexical processing, therefore, lexical recognition and lexicon selection played a dominant role across conditions regardless of spatial processing demand.

A recently proposed “interactive account” suggested that the ventral occipito-temporal region acts as an interface linking visual word form processing with processing of other aspects in both bottom-up and top-down directions [26], [50]. In the current study, FG showed the greatest activation to real characters and received top-down modulations from SPL during both spatial and lexical task requirements. Therefore, consistent with the interactive account, the current study provides evidence of effective connectivity, indicating top-down modulation from the posterior parietal region to the ventral occipito-temporal region during visual lexical recognition, and suggesting that the ventral pathway plays a dominant role in lexical recognition.

Behavioral performance showed that conditions requiring radical rearrangement (RTC and RPC) were more difficult, which could also affect SPL activation. So these influences seem to be inevitable and predictable due to the current experimental design. Further studies should attempt to control this potential confound. In addition, as previously mentioned, tasks in current study less relied on phonological processing, so future studies could explore the effect of phonological processing demand on the current network.

### Conclusion

By taking advantage of the unique qualities of Chinese characters, the current study investigated interactions between the dorsal and ventral pathways in lexical recognition and explored how these connections could be modulated by lexical and spatial demands. Effective connectivity results revealed that there is an intrinsic connection from the left superior parietal lobule (dorsal pathway) to the left fusiform gyrus (ventral pathway), which is modulated by both spatial and lexical demands. Regions in the dorsal stream receive modulation from the prefrontal region, and in turn, modulate the ventral stream to assist in visual recognition of words. Taken together, the current study suggests that the dorsal stream is highly involved in lexical recognition and acts as a top-down modulator for lexical selection.

## Supporting Information

Table S1 **Brain regions showing significant activations for each comparison.**
(DOC)
