# Supplementary material for: Top-Down Modulations from Dorsal Stream in Lexical Recognition: An Effective Connectivity fMRI Study
Source: PLoS One. 2012 Mar 13;7(3):e33337. doi: 10.1371/journal.pone.0033337 (PMC3302835; doi:10.1371/journal.pone.0033337)
Supplement: Table S1 — Brain regions showing significant activations for each comparison. (DOC) [file pone.0033337.s001.doc]

**Table S1. Brain regions showing significant activations for each comparison.**

| Activated regions | BA | Voxels | Z value | x,y,z {mm} |
| --- | --- | --- | --- | --- |
| *TC> RTC ** |  |  |  |  |
| L precentral gyrus | 4/5 | 51 | 3.75 | -34 -30 62 |
|  |  |  |  |  |
| *TC> RPC* |  |  |  |  |
| L postcentral gyrus | 6/4 | 351 | 4.29 | -44 -26 58 |
| L precentral gyrus | 44 | 39 | 3.87 | -62 8 12 |
| L fusiform gyrus | 18/19 | 31 | 3.26 | -28 -86 -20 |
| R middle occipital gyrus | 18 | 47 | 4.08 | 36 -82 -16 |
|  |  |  |  |  |
| *RTC> TC* |  |  |  |  |
| L inferior frontal gyrus | 44/9 | 192 | 3.52 | -44 4 22 |
| L **insula** / inferior frontal gyrus | 13/47 | 114 | 3.11 | -30 18 4 |
| L **medial frontal gyrus** / superior frontal gyrus | 6/8 | 659 | 4.43 | -10 18 48 |
| L middle frontal gyrus | 6 | 68 | 3.55 | -24 -2 52 |
| L **middle temporal gyrus /** middle occipital gyrus / fusiform / superior parietal lobule / inferior parietal lobule | 19/37/7/40 | 3691 | 4.33 | -38 -54 -24 |
| L putamen | - | 249 | 3.64 | -14 12 -4 |
| L thalamus | - | 201 | 4.49 | -8 -12 -10 |
| L lentiform nucleus | - | 27 | 2.96 | -16 -8 2 |
| R **inferior frontal gyrus** /insula | 45/47/13 | 171 | 4.06 | 30 26 10 |
| R **inferior frontal gyrus** / middle frontal gyrus | 9 | 219 | 3.88 | 48 6 32 |
| R inferior parietal loblue | 40 | 124 | 4.05 | 34 -44 36 |
| R **middle temporal gyrus** / middle occipital gyrus / fusiform | 19/37 | 2672 | 5.21 | 38 -84 16 |
| R precuneus | 7/19 | 12 | 3.14 | 30 -72 34 |
| R **precunues** / superior parietal lobule | 7 | 465 | 4.12 | 28 -66 60 |
| R anterior cingulate | 24 | 15 | 2.44 | 4 8 28 |
| R thalamus | - | 233 | 4.3 | 10 -16 0 |
| R putamen | - | 182 | 4.17 | 12 8 4 |
| R **culmen** / L culmen | - | 125 | 4.11 | 2 -62 -30 |
| R **declive** / L declive | - | 188 | 3.96 | -6 -74 -30 |
| R uvula | - | 19 | 3.84 | 8 -80 -42 |
|  |  |  |  |  |
| *RPC> TC* |  |  |  |  |
| L inferior frontal gyrus | 9 | 111 | 3.24 | -46 8 32 |
| L middle frontal gyrus | 9 | 62 | 3.65 | -44 22 32 |
| L middle frontal gyrus | 6 | 27 | 3.24 | -24 -2 54 |
| L middle occipital gyrus | 19/37 | 1631 | 3.58 | -40 -90 14 |
| L **superior parietal lobule** / precuneus | 7 | 652 | 3.7 | -22 -60 56 |
| L **putamen** /insula | -/13 | 353 | 4.29 | -12 6 -2 |
| L **culmen** / declive / R declive | - | 408 | 5.39 | -6 -60 -14 |
| R **inferior frontal gyrus** / insula | 45/13 | 211 | 3.78 | 36 26 6 |
| R **inferior frontal gyrus** / middle frontal gyrus | 9 | 80 | 3.17 | 48 6 32 |
| R medial frontal gyrus | 9 | 15 | 3.07 | 6 32 38 |
| R **medial frontal gyrus** / superior frontal gyrus / cingulate gyrus | 6/8/24 | 673 | 3.61 | 8 14 52 |
| R middle occipital gyrus | 19 | 1076 | 3.55 | 48 -78 6 |
| R **precentral gyrus** / postcentral gyrus | 4,3 | 780 | 4.93 | 44 -22 62 |
| R **precuneus** / superior parietal loblue | 7 | 395 | 3.9 | 24 -66 40 |
| R putamen | - | 22 | 3.69 | 18 6 -8 |
| R putamen | - | 32 | 3.58 | 16 6 2 |
| R **thalamus**/L thalamus | - | 964 | 4.78 | 12 -16 0 |
|  |  |  |  |  |
| *RTC> RPC* |  |  |  |  |
| - | - | - | - | - |
|  |  |  |  |  |
| *RPC> RTC* |  |  |  |  |
| L middle frontal gyrus | 46/9 | 12 | 3.35 | -44 24 28 |
| L declive | - | 24 | 3.11 | -10 -56 -16 |
| R **postcentral gyrus** / middle frontal gyrus | 3/6 | 523 | 5.27 | 42 -24 54 |
| R declive | - | 15 | 3.02 | 6 -74 -28 |
| R subthalamic nucleus | - | 85 | 3.78 | 12 -24 -6 |

L, left hemisphere; R, right hemisphere; BA, Brodmann’s areas. Areas in boldface indicate peaks of activation in the clusters. TC, true character; RTC, radical-rearranged true characters; RPC, radical-rearranged pseudo-characters. Significance at *p* < 0.05, FDR corrected (*Significance at *p* < 0.001, uncorrected).
